# Oral alkalinizing supplementation suppressed intrarenal reactive oxidative stress in mild-stage chronic kidney disease: a randomized cohort study

**DOI:** 10.1007/s10157-024-02517-3

**Published:** 2024-06-13

**Authors:** Michiaki Abe, Takuhiro Yamaguchi, Seizo Koshiba, Shin Takayama, Toshiki Nakai, Koichiro Nishioka, Satomi Yamasaki, Kazuhiko Kawaguchi, Masanori Umeyama, Atsuko Masaura, Kota Ishizawa, Ryutaro Arita, Takeshi Kanno, Tetsuya Akaishi, Mariko Miyazaki, Takaaki Abe, Tetsuhiro Tanaka, Tadashi Ishii, CKOALA study group

**Affiliations:** 1 Department of Education and Support for Regional Medicine, Tohoku University Hospital, 1-1 Seiryo-Machi, Sendai, Miyagi 9808574 Japan; 2 Tohoku Medical Megabank Organization, Tohoku University, Sendai, Miyagi Japan; 3 Department of Nephrology, Rheumatology and Endocrinology, Tohoku University Graduate School of Medicine, Sendai, Miyagi Japan; 4 Clinical Research Data Center, Tohoku University Hospital, Sendai, Miyagi Japan; 5 Medical Affairs Department, Nippon Chemiphar Co., Ltd, Chiyoda-Ku, Tokyo, Japan; 6 Development Planning Department, Nippon Chemiphar Co., Ltd, Chiyoda-Ku, Tokyo, Japan; 7 Department of Clinical Biology and Hormonal Regulation, Tohoku University Graduate School of Medicine, Sendai, Miyagi Japan

**Keywords:** Citrate, Bicarbonate, Chronic kidney disease, Reactive oxygen species, Metabolic acidosis, Aciduria

## Abstract

**Background:**

The beneficial effects of oral supplements with alkalinizing agents in patients with chronic kidney disease (CKD) have been limited to the severe stages. We investigated whether two types of supplements, sodium bicarbonate (SB) and potassium citrate/sodium citrate (PCSC), could maintain renal function in patients with mild-stage CKD.

**Methods:**

This was a single-center, open-labeled, randomized cohort trial. Study participants with CKD stages G2, G3a, and G3b were enrolled between March 2013 and January 2019 and randomly assigned by stratification according to age, sex, estimated glomerular filtration rate (eGFR), and diabetes. They were followed up for 6 months (short-term study) for the primary endpoints and extended to 2 years (long-term study) for the secondary endpoints. Supplementary doses were adjusted to achieve an early morning urinary pH of 6.8–7.2. We observed renal dysfunction or new-onset cerebrovascular disease and evaluated urinary surrogate markers for renal injury.

**Results:**

Overall, 101 participants were registered and allocated to three groups: standard (*n* = 32), SB (*n* = 34), and PCSC (*n* = 35). Two patients in the standard group attained the primary endpoints (renal stones and overt proteinuria) but were not statistically significant. There was one patient in the standard reduced eGFR during the long-term study (*p* = 0.042 by ANOVA). SB increased proteinuria (*p* = 0.0139, baseline vs. 6 months), whereas PCSC significantly reduced proteinuria (*p* = 0.0061, baseline vs. 1 year, or *p* = 0.0186, vs. 2 years) and urinary excretion of 8-hydroxy-2′-deoxyguanosine (*p* = 0.0481, baseline vs. 6 months).

**Conclusion:**

This study is the first to report supplementation of PCSC reduced intrarenal oxidative stress in patients with mild-stage CKD.

**Graphical abstract:**

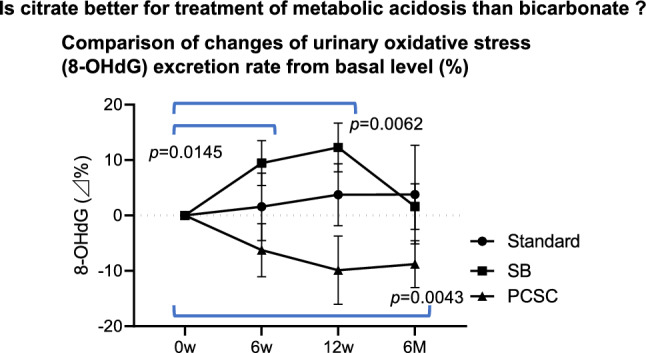

**Supplementary Information:**

The online version contains supplementary material available at 10.1007/s10157-024-02517-3.

## Introduction

Aciduria is caused by the urinary excretion of acid waste and uremic toxins produced on a daily basis. Metabolic acidosis is a result of renal excretory dysfunction and accumulation of waste and uremic toxins in patients with chronic kidney disease (CKD). Metabolic acidosis is a risk factor for CKD progression and cardiovascular diseases (CVD) [1]. Alkali loads can neutralize metabolic acidosis; however, the reno-protective effects of supplements in patients with CKD are thought to be limited because exogenic alkali loads are excreted very quickly in the urine [2]. Additionally, whether exogenic alkali loads affect renal function remains controversial. Several randomized controlled trials (RCT) on patients with CKD and metabolic acidosis indicated that the chronic administration of sodium bicarbonate (SB) neutralized metabolic acidosis and slowed the decline rate of kidney function [3–7]. One double-blinded RCT study suggested that SB slowed the estimated glomerular filtration rate (eGFR) decline in mild-CKD stages of hypertensive nephropathy [8]; however, other double-blinded RCT studies have not indicated the reno-protective effects in the mild-stages of CKD [9–11]. The effect of alkalinizing supplementation on mild-stage CKD remains controversial. One study reported that another alkalinizing supplement, sodium citrate (SC), has reno-protective effects against hypertensive nephropathy [12]. However, RCTs on the effects of oral citrate supplementation in CKD have not yet been conducted.

We hypothesized that chronic oral alkalinizing supplementation could preserve the renal function in mild-stage CKD by monitoring the urinary pH and evaluating surrogate markers, including the intrarenal reactive oxygen species (ROS), to predict the associated renal dysfunction.

## Methods

### Study design and patient registration

This single-center, open-label, randomized cohort trial was named Oral ALkalizers in patients with Chronic Kidney disease (CKOALA study). The detailed study protocol has been published previously [13]. Two oral alkalinizing supplements, SB and potassium citrate/sodium citrate (PCSC), were used. Metabolic acidosis is defined as a serum bicarbonate level < 22 mEq/L with normal respiratory function, which is usually recognized in the severe stages of CKD with hyperpotassemia [14]. Therefore, we enrolled patients with mild-stage CKD to avoid the severe adverse effects of hyperkalemia because the PCSC included potassium. Patients aged 20–80 years with CKD stages G2, G3a, and G3b were recruited at the Tohoku University Hospital. The registered patients were layered using four variables: age (≥ 65, < 65 years old), sex, presence of diabetes mellitus, and estimated creatinine glomerular filtration rate (eGFR), ≥ 46 or < 46 mL/min/1.73 m^2^). All patients were randomly allocated to three groups using a computer method: standard, SB, and PCSC. Sex was self-reported during the first visit. SB or PCSC was started at 1.5 g per day. These drugs have been approved for the treatment of acidosis and hyperuricemia in Japan. When the early morning urinary pH (mUpH) was < 6.5, the agent dose was increased to 3.0 g per day. When the early mUpH was > 7.2, the dose was decreased to that of mUpH < 6.5. mUpH was measured using a urinary pH meter (LAQUAtwin pH sensor S010, HORIBA Ltd., Kyoto, Japan). All the registered patients provided written informed consent. The patients were followed up at baseline, 6 weeks (6W), 12 weeks (12W), and 6 months (6 M) for the short-term study. Patients who completed the short-term study were reconsented at 6 months to continue the long-term study for an additional 1 year (1Y) to 2 years (2Y). The exclusion criteria were as follows: eating and drinking abundant amounts of alkalinizing substances, taking tolvaptan, renal hypouricemia, hyperkalemia, diabetes insipidus, hypernatremia of unknown origin, untreated mUpH > 6.8, serious urinary tract infection, and serious complications of heart and liver disease.

### Data collection and sample assay

The performance status, venous blood tests, spot urine tests, venous blood gas tests, serum creatinine (sCr), eGFR of rate (eGFR-Cr) of creatinine, proteinuria (UP), urinary excretion of albumin (UAE), and N-acetyl-beta-d-glucosaminidase (UNAG) were performed at the central laboratory of Tohoku University Hospital. For other urinary surrogate biomarkers, alfa1-microglobulin (UaMG, mg/L) and type IV collagen (U4Col) were measured at the SRL Laboratory (SRL, Inc., Tokyo, Japan). L-type fatty acid binding protein (ULFABP), neutrophil gelatinase-associated lipocalin (UNGAL), kidney injury molecule-1 (UKIM-1), transforming growth factor-beta (UTGFb), endothelin-1 (UET-1), angiotensinogen (UANG), monocyte chemotactic protein-1 (UMCP-1), interleukin-6 (UIL-6), aldosterone (UAldo) and lactate (ULac) were measured at Safety Research Institute for Chemical Compounds Co., Ltd. (Sapporo, Japan). For intrarenal ROS assays, 8-isoprostane (U8IsoP) and 8-hydroxy-2′-deoxyguanosine (U8OHdG) were measured at NIKKEN SEIL Co., Ltd. (Shizuoka, Japan). The Health Related Quality of Life Short Form 8TM Health Survey © (SF8) was licensed to the Institute for Health Outcomes and Process Evaluation research (iHope International, Kyoto, Japan) for the evaluation of individual performances. The renal function of eGFR was calculated using the following equation: eGFR-Cr = 194 × Cr^−1.094^ × age^−0.287^ for men, or 194 × Cr^−1.094^ × age^−0.287^ × 0.739 for women [15]. All samples at every visit were stored at –80 °C for subsequent analysis of urinary surrogate biomarkers.

### Endpoints

The primary endpoints were the development of significant renal dysfunction as follows; (1) sCr level ≥ 1.5 × higher than that of baseline, (2) eGFR decrease ≥ 20 mL/min/1.73 m^2^ from baseline, (3) UP ≥ 3.5 g/gCr (overt), and (4) new development of urinary stones or the occurrence of CVD during the short-term study. The secondary endpoints were the same items as the primary endpoints at 1 and 2 years and the exploratory research of surrogate biomarkers associated with the reno-protective effects of the interventions.

### Statistical analysis

The ideal estimated sample size of 50 participants in each group was to achieve the level of significance (*i.e.* type-1 error rate) of 5% (α = 0.05) and the statistical power of 80% (β = 0.2) with the equally allocated three groups referring to previous studies [3] as described previously [16]. However, two earlier separate studies of patients suggested the need for 33–36 patients in each of the three groups to achieve sufficient power to detect the eGFR benefit of the interventions by SB, SC, or fruits and vegetables (FV) among the groups compared with the control group [7, 12]. Therefore, we changed the registration period to 5 years, and the target number was approximately 35 for each group; the modified protocol was also approved by the Ethics Committee.

For the intention-to-treat analysis, the collection and management of the data was conducted by an individual data management team at the Clinical Research Data Center of Tohoku University Hospital. First, the appearance rates of primary endpoints at 6W, 12W, and 6 M during each treatment were compared with the standard (Student’s *t*-test). Second, changes in the renal function (sCr, eGFR-Cr, and UP) were compared using a paired *t*-test between each visit value and baseline for each group, or the Wilcoxon test among the three groups at each visit. An analysis of variance (ANOVA) was used for the secondary endpoints. Changes in other urinary surrogate markers and quality of life with SF-8 were analyzed using a paired *t*-test or Wilcoxon test using the same method. The significance of comparison among the three groups was *p* < 0.0167 for adapting Bonferroni correction, and the other was *p* < 0.05.

Efficacy was analyzed for the intention-to-treat population, that is the group of randomized subjects who received at least one dose of the study drug and underwent at least one post-drug efficacy assessment. Safety was analyzed in all patients who received at least one dose of the study drug. All statistical analyses were performed using Windows SAS software (version 9.4; SAS Institute, Cary, NC, USA).

### Ethical matters

The trial was conducted in accordance with the Good Clinical Practice guidelines of the International Council for Harmonization and the ethical principles of the Declaration of Helsinki. Written informed consent was obtained from all patients. The Ethics Committee of Tohoku University Hospital approved this trial protocol (IRB2012-2–100-1 and CRB2200003). All registered data were monitored by independent central reviewers throughout the study.

### Trial registration

The trial was registered on February 26, 2013 (UMIN-CTR 000010059), and March 26, 2019 (jRCTs 021180043).

## Results

### Patients and basal characteristics

A total of 101 patients [age (mean ± SD) 61.6 ± 11.5 years] with CKD stages G2, G3a, and G3b were registered between April 2013 and March 2018. As shown in Figure, the patients were allocated into three groups: standard (*n* = 31), SB (*n* = 32), and PCSC (*n* = 32). Six registered patients withdrew consent due to research anxieties or surgeries for worsened comorbidities before starting administration (Standard 1, SB 2, and PCSC 3). Four patients dropped out during the short-term study period (SB 1 and PCSC 3). In a long-term study, only 29 patients reconsented at 6 M (standard 4, SB 12, and PCSC, 13). The most common reason for this disagreement is the desire to consume alkalinizing supplements. Four patients dropped out after 2Y (standard 1, SB 2, and PCSC 1). No severe intervention-related adverse events were observed.

No differences in age, sex, medications, medical history, or malignancies that developed within 5 years were observed among the three groups (Table 1). The baseline physiological findings and blood and urine examinations were not significantly different among the three groups (Table 2).

**Table 1:** Background of age, sex, existing diseases and medication of the participants

	Standard (*n* = 31)	SB (*n* = 32)	SPC (*n* = 32)	*p* value*
Sex				
Male	21 (68%)	21 (66%)	21 (66%)	0.979
Female	10 (32%)	11 (34%)	11 (34%)	
Age category				
≤ 65 years old	17 (55%)	17 (53%)	17 (53%)	0.988
> 65 years old	14 (45%)	15 (47%)	15 (47%)	
Disease				
Hypertension	9 (29%)	10 (31%)	12 (38%)	0.7575
Dyslipidemia	3 (10%)	4 (13%)	3 (9%)	0.9043
Cerebrovascular	4 (13%)	7 (22%)	4 (13%)	0.5102
Heart disease	4 (13%)	10 (31%)	8 (25%)	0.2154
COPD	1 (3%)	4 (13%)	5 (16%)	0.2503
Glomerulonephritis	9 (29%)	3 (9%)	8 (25%)	0.1279
PAS	1 (3%)	1 (3%)	1 (3%)	0.9997
Malignancy	3 (10%)	2 (6%)	2 (6%)	0.8355
Drug				
Aldosterone inhibitors	8 (26%)	4 (13%)	5 (16%)	0.3558
Alfa blocker	6 (19%)	2 (6%)	5 (16%)	0.2947
Anti RAS	17 (55%)	15 (47%)	14 (44%)	0.6632
Antiplatelet	13 (42%)	9 (28%)	9 (28%)	0.4042
Alfa GI	4 (13%)	4 (13%)	4 (13%)	0.9985
Beta blocker	10 (32%)	8 (25%)	7 (22%)	0.6317
BG	1 (3%)	6 (19%)	3 (9%)	0.1289
Bisphosphonate	2 (6%)	1 (3%)	2 (6%)	0.801
Bronchodilators	3 (10%)	3 (9%)	4 (13%)	0.9043
CCB	12 (39%)	15 (47%)	15 (47%)	0.7541
DPP4 inhibitor	6 (19%)	3 (9%)	7 (22%)	0.3693
ESA	0 (0%)	0 (0%)	2 (6%)	0.1338
Fe drug	1 (3%)	0 (0%)	3 (9%)	0.1655
Fibrate	3 (10%)	3 (9%)	1 (3%)	0.5286
FK506	1 (3%)	0 (0%)	1 (3%)	0.595
Glinid	3 (10%)	3 (9%)	1 (3%)	0.5286
GLP1	0 (0%)	1 (3%)	0 (0%)	0.3698
Insulin	2 (6%)	1 (3%)	2 (6%)	0.801
Loop diuretics	0 (0%)	0 (0%)	3 (9%)	0.0474*
NPC1L1 blocker	5 (16%)	3 (9%)	0 (0%)	0.0683
NSAID	2 (6%)	1 (3%)	3 (9%)	0.5893
Omeg3	2 (6%)	7 (22%)	3 (9%)	0.1453
PPI	4 (13%)	5 (16%)	3 (9%)	0.7522
RANKL inhibitor	0 (0%)	1 (3%)	0 (0%)	0.3698
Sgl2 inhibitor	1 (3%)	1 (3%)	1 (3%)	0.9997
Statin	10 (32%)	11 (34%)	7 (22%)	0.503
Steroid	1 (3%)	1 (3%)	4 (13%)	0.2102
SU	0 (0%)	1 (3%)	1 (3%)	0.6097
Thiazide	1 (3%)	7 (22%)	7 (22%)	0.0651
UA excretion drugs	3 (10%)	7 (22%)	2 (6%)	0.142
UA synthase inhibitor	15 (48%)	13 (41%)	12 (38%)	0.6673
Vitamin D	3 (10%)	2 (6%)	2 (6%)	0.8355

^*^p < 0.0167 for the significance

*COPD* chronic obstructive pulmonary disease; *PAS* peripheral atherosclerosis; *RAS* renin-angiotensin system; *GI* glucosidase inhibitor; *BG* biguanide; *CCB* calcium-channel blocker; *DPP4* dipeptidyl peptidase 4; *ESA* erythropoiesis stimulating agent; *GLP1* glucagon-like peptide 1; *NPC1L1* Niemann-Pick C1-like 1; *NSAID* non-steroidal anti-inflammatory drug; *omeg3* omega-3 fatty acids; *PPI* proton pump inhibitor; *RANKL* receptor activator of nuclear factor-kappa B ligand; *Sgl2* sodium-glucose cotransporter 2; *SU* sulfonylurea; *UA* uric acid

**Table 2:** Basal characteristics of physiological findings and blood and urine examination on renal function

0w	ALL			Standard			SB			SPC			*p* value*
	*n*	Median	IQ range (Q1, Q3)	*n*	Median	IQ range (Q1, Q3)	*n*	Median	IQ range (Q1, Q3)	*n*	Median	IQ range (Q1, Q3)	
Number	95			31			32			32			
Sex (female)	33			10			11			11			0.9944
Age, years old	95	65	55–70	31	65.0	58, 68	32	65.0	52.3, 70	32	64.0	56.5, 70.8	0.9947
≥ 65	51	70	67, 73	18	68	66, 74.3	17	70	67, 72	16	71	67, 74	0.3817
Physical findings													
Body Height, cm	95	163.5	158.5, 169	31	163.3	155.1, 168.2	32	163.7	159.2, 169.4	31	163.2	158.9, 169.4	0.8287
Body Weight, kg	95	71.5	59.8, 81.5	31	72.9	55, 79.6	32	71.4	58.6, 83.0	32	69.0	60.2, 82.5	0.8842
BMI	95	26.3	22.8, 29.5	31	26.2	23.7, 29.1	32	26.9	23.7, 30.2	32	26.0	22.2, 30.0	0.7373
SBP, mmHg	95	128	119, 139	31	128.0	117, 142	31	132.0	123, 143	32	126.0	115, 134.5	0.1698
DBP, mmHg	95	80	70, 87	31	82.0	78, 86	31	77.0	69, 88	32	74.0	68.3, 85.3	0.2371
Pulse, bpm	95	50	66, 80	31	70.0	64, 79	31	72.0	66, 84	32	76.0	68, 79	0.5458
Blood examinations													
WBC, × 10^3^/uL	95	5.9	4.8, 7	31	5.7	4.8, 6.5	32	6.2	5.1, 7	32	5.8	4.6, 7.4	0.715
RBC, × 10^6^/uL	95	4.62	4.3, 5	31	4.6	4.4, 5.2	32	4.6	4.3, 4.9	32	4.7	4.1, 5.2	0.5986
Hb, g.dL	95	14.2	13.4, 15.5	31	14.2	13.7, 15.4	32	14.2	13.4, 15.1	32	14.4	12.6, 16.4	0.9823
Ht, %	95	42.5	39.9, 46.4	31	42.8	40.6, 46.6	32	41.6	39.7, 45.3	32	43.6	37.4, 47.3	0.7084
Plt, 10^3^/uL	95	212	189, 262	31	229.0	191, 268	32	220.5	191, 269.5	32	200.0	174.5, 240.5	0.0968
Reticulocyte, ‰	91	1.4	1.2, 1.7	29	1.4	1.2, 1.4	30	1.5	1.3, 2	32	1.4	1.2, 1.6	0.9648
BUN, mg/dL	95	17	15, 22	31	19.0	15, 23	32	16.0	14.3, 20	32	16.5	14.3, 22	0.3432
Cr, mg/dL	95	1.01	0.84, 1.20	31	1.01	0.81, 1.43	32	0.99	0.8, 1.13	32	1.03	0.91, 1.22	0.5798
UA, mg/dL	95	5.9	5, 6.8	31	6.2	5, 6.9	32	5.4	5, 6.65	32	6.0	5.15, 6.8	0.4244
TP, g/dL	94	7.2	6.9, 7.5	31	7.2	7.0, 7.5	32	7.2	7.0, 7.4	32	7.1	6.9, 7.7	0.7828
Alb, g/dL	94	4.2	4.1, 4.4	31	4.2	4.0, 4.4	31	4.2	4.1, 4.4	32	4.1	4.1, 4.4	0.5699
Na, mEq/L	95	140	139, 141	31	139.0	138, 141	32	140.5	139.3, 141	32	140.0	139.3, 142	0.1297
K, mEq/L	95	4.3	4.1, 4.6	31	4.4	4.2, 4.6	32	4.3	4, 4.4	32	4.3	4.1, 4.6	0.1103
Cl, mEq/L	95	104	103, 106	31	104	102, 107	32	104	103, 105	32	105	104, 106.8	0.0237
Ca, mg/dL	95	9.3	9.1, 9.6	31	9.2	9.0, 9.6	32	9.4	9.1, 9.6	32	9.3	9.0, 9.6	0.912
IP, mg/dL	95	3.2	2.8, 3.6	31	3.2	2.7, 3.6	32	3.2	2.8, 3.6	32	3.4	3.0, 3.7	0.4157
Glucose	95	106	96, 118	31	104.0	92, 121	32	107.5	96.3, 122	32	106.5	97, 115	0.7006
HbA1cNGS	94	5.9	5.7, 6.2	31	6.0	5.7, 6.2	31	5.9	5.6, 6.2	32	5.9	5.6, 6.2	0.8734
HCO3-, mEq/L	94	24.85	23.5, 26.5	31	25.4	24.2, 26.9	31	25.4	23.5, 26.8	32	24.2	23.2, 26.2	0.1864
aBE, mEq/L	89	0.1	-1, 1.15	30	0.3	-0.75, 1.25	30	0.4	-0.63, 1.53	29	-0.3	-1.7, 0.73	0.0616
eGFR-Cr, mL/min/1.73m^2^	95	55.22	43.27–63.4	31	54.8	38.0, 64.7	32	55.8	46.9, 64.8	32	56.1	43.3, 61.5	0.6587
Urine examinations													
UpH	93	5.83	5.48–6.19	31	5.7	5.4, 6.2	32	5.9	5.5, 6.3	32	5.9	5.5, 6.2	0.715
UP, g/gCr	91	0.1	0.03, 0.23	31	0.1	0.05, 0.18	31	0.1	0.05, 0.25	31	0.1	0.05, 0.32	0.9318
UAE, mg/gCr	91	24.4	7.1, 159.7	31	18.7	8, 111.8	29	17.9	5.3, 175.9	31	42.9	7.1, 301.4	0.7814
Health QOL													
SF8	94	15.5	13–19	31	16.0	12, 19	32	14.5	11.5, 19	31	16.0	14, 20	0.8687

^*^p < 0.0167 for the significance

*IQ* Internal quartile; *Q1* first quartile; *Q3* third quartile; *BMI* body mass index; *SBP* systolic blood pressure; *DBP* diastolic blood pressure; *WBC* white blood cell; *RBC* red blood cell; *Hb* hemoglobin; *Ht* hematocrit; *Plt* platelet; *BUN* blood urea nitrogen; *Cr* creatinine; *UA* uric acid; *TP* total protein; *Alb* albumin; *aBE* actual base excess; *eGFR-Cr* estimated creatinine glomerular filtration rate of creatinine; *UpH* urinary pH; *UP* proteinuria; *UAE* urinary excretion of albumin

### Primary endpoints

There was no significant difference in the sCr and eGFR-Cr among the three groups at 6 months [sCr (g/dL, mean ± SE); Standard 1.13 ± 0.06, SB 0.98 ± 0.04, PCSC 1.08 ± 0.05: eGFR-Cr (mL/min/1.73 m^2^); Standard 52.0 ± 2.84, SB 58.1 ± 2.35, PCSC 52.8 ± 2.10]. None of the groups showed a significant increase in renal function from the baseline (Table 3).

**Table 3:** Upper: number of achievement of primary endpoints and secondary endpoints. Lower: Performance status of SF-8 by the interventions at 6 M

Achievement of primary endpoints					
		*n*	Yes	No	*p* value, vs. Standard
6W					
	Standard	29	1	29	
	SB	27	0	27	1
	PCSC	30	0	30	0.65
12W					
	Standard	31	1	30	
	SB	31	0	31	1
	PCSC	29	0	29	1
6M					
	Standard	31	2	29	
	SB	31	0	31	0.55
	PCSC	29	0	31	0.55
Achievement of secondary endpoints					
		*N*	Yes	No	*p* value*, vs. Standard
1Y					
	Standard	5	1	4	
	SB	6	0	6	0.24
	PCSC	10	0	10	0.24
2Y					
	Standard	3	1	3	
	SB	4	0	4	1
	PCSC	9	0	9	0.24

SF-8									
	Standard			SB			PCSC		
	Mean	SD	SE	Mean	SD	SE	Mean	SD	SE
GH	49.4	40.1	7.2	50.1	38.4	6.9	52.8	28.8	5.1
PF	49.2	28.4	5.1	48.6	37.3	6.7	50.2	33.4	5.9
RP	50.9	23.4	4.2	49.4	41.8	7.5	50.4	30	5.3
BP	53.3	41.8	7.5	52.7	47.3	8.5	54	41.3	7.3
VT	48.4	31.2	5.6	50.5	39	7	49.4	47	8.3
SF	51.1	36.7	6.6	48	55.7	10	49.4	47	8.3
MH	52.2	30.6	5.5	50.6	45.1	8.1	51.9	33.4	5.9
RE	51.5	22.8	4.1	48.9	40.6	7.3	49.7	34.5	6.1

Values were described as mean, standard deviation (SD), and standard error (SE). There were no statistically significant differences in any items between the other two groups compared to the standard group in these two panels

*SB* sodium bicarbonate; *PCSC* potassium citrate/sodium citrate; *GH* general health; *PF* physical functioning; *RP* role physical; *BP* bodily pain; *VT* vitality; *SF* social functioning; *MH* mental health; *RE* role emotional

One patient experienced overt UP after 6 weeks compared with that at baseline (from 3.17 g/gCr to 4.02), and one patient developed newly onset renal stones at 6 months in the standard group; however, renal dysfunction was not significantly different among the three groups (Tables 3 and 4). No new CVD complications occurred.

**Table 4:** Outcomes of secondary endpoints of urinary surrogate markers of each visit

			Baseline			6W			12W			6 M			1Y			2Y		
			Mean	SD	SE	Mean	SD	SE	Mean	SD	SE	Mean	SD	SE	Mean	SD	SE	Mean	SD	SE
sCr	g/gL																			
	Standard		1.089	0.34	0.06	1.096	0.316	0.056	1.087	0.334	0.059	1.13	0.351	0.062	1.232	0.194	0.097	1.132	0.130	0.075
	*p* value*, vs. baseline					0.269			0.6411			0.5418			0.056			0.7452		
	SB		0.994	0.239	0.042	1.001	0.219	0.036	0.992	0.235	0.041	0.981	0.237	0.041	0.987	0.127	0.048	0.969	0.098	0.044
	*p* value*, vs. baseline					0.1675			0.5473			0.1938			0.7437			0.3394		
	PCSC		1.054	0.246	0.043	1.098	0.265	0.048	1.067	0.239	0.046	1.075	0.251	0.048	1.061	0.171	0.054	1.079	0.174	0.058
	*p* value*, vs. baseline					0.0612			0.5128			0.3844			0.7971			0.557		
	*p* value among 3 groups**					0.1817			0.3121			0.0989			0.0772			0.1176		
	*p* value by ANOVA*		0.0166*																	
			baseline			6W			12W			6 M			1Y			2Y		
			mean	SD	SE	mean	SD	SE	mean	SD	SE	mean	SD	SE	mean	SD	SE	mean	SD	SE
eGFR-Cr	mL/min/1.73 m^2^																			
	Standard		53.94	15.06	2.66	52.87	13.84	2.42	54.25	16.45	2.91	52.01	16.05	2.84	47.92	9.000	4.5	51.81	5.127	2.96
	*p* value*, vs. baseline					0.7781			0.7627			0.4122			0.0402*			0.9234		
	SB		57.53	13.65	2.38	56.27	12.64	2.05	58.05	15.75	2.72	58.12	13.64	2.35	56.63	6.667	2.52	56.8	4.696	2.1
	*p* value*, vs. baseline					0.8818			0.6186			0.3274			0.2691			0.4118		
	PCSC		53.41	10.78	1.88	51.95	12.85	2.3	53.24	12.11	2.23	52.75	11.51	2.1	55.22	8.601	2.72	51.07	7.080	2.36
	*p* value*, vs. baseline					0.1502			0.8717			0.539			0.2487			0.142		
	*p* value among 3 groups**					0.3309			0.3817			0.1533			0.2406			0.1551		
	*p* value by ANOVA*		0.0420																	
			Baseline			6W			12W			6 M			1Y			2Y		
			Mean	SD	SE	Mean	SD	SE	Mean	SD	SE	Mean	SD	SE	Mean	SD	SE	Mean	SD	SE
UP	g/gCr																			
	Standard		0.271	0.577	0.1	0.331	0.8	0.14	0.33	0.729	0.13	0.367	0.731	0.13	0.192	0.074	0.07	0.241	0.066	0.09
	*p* value*, vs. baseline					0.131			0.2538			0.0594			0.6369			0.3713		
	SB		0.252	0.418	0.07	0.31	0.545	0.09	0.359	0.605	0.11	0.421	0.697	0.12	0.162	0.087	0.05	0.256	4.001	0.06
	*p* value*, vs. baseline					0.0883			0.1232			0.0139*			0.7251			0.0555		
	PCSC		0.306	0.509	0.09	0.272	0.514	0.09	0.28	0.527	0.09	0.287	0.486	0.09	0.198	0.004	0.05	0.241	0.068	0.07
	*p* value*, vs. baseline					0.4307			0.6465			0.678			0.0061*			0.0186*		
	*p* value among 3 groups**					0.9234			0.8463			0.6463			0.8743			0.9841		
	*p* value by ANOVA*		0.2443																	
			Baseline			6W			12W			6 M			1Y			2Y		
			Mean	SD	SE	Mean	SD	SE	Mean	SD	SE	Mean	SD	SE	Mean	SD	SE	Mean	SD	SE
UaMG	mg/L																			
	Standard		4.0365	5.731	1.0126	3.8167	5.704	1.0239	4.7	10.787	1.9364	4.35	7.932	1.4415	5.9333	5.556	2.6206	3.9667	2.845	1.3411
	*p* value*, vs. baseline					0.4671			0.3336			0.4306			0.9192			0.6227		
	SB		3.1625	2.808	0.4886	3.6037	3.685	0.696	3.671	3.668	0.6482	3.8968	3.409	0.6024	1.4333	0.252	0.1186	2.4667	2.021	0.9526
	*p* value*, vs. baseline					0.1884			0.2411			0.1826			0.0989			0.8221		
	PCSC		3.3094	3.870	0.6734	2.5233	2.509	0.4505	2.7207	2.901	0.5294	2.8586	2.829	0.5162	5.58	5.887	2.3548	2.32	1.264	0.5055
	*p* value*, vs. baseline					0.2352			0.3884			0.5058			0.3367			0.243		
	*p* value among 3 groups**					0.2889			0.38			0.333			0.0509			0.5169		
	*p* value by ANOVA*		0.1067																	
			Baseline			6W			12W			6 M			1Y			2Y		
			Mean	SD	SE	Mean	SD	SE	Mean	SD	SE	Mean	SD	SE	Mean	SD	SE	Mean	SD	SE
U4Col	μg/gCr																			
	Standard		3.6935	4.661	0.8294	4.5083	7.019	1.2683	3.4933	2.882	0.5216	2.8532	1.567	0.2916	1.825	0.772	0.3342	10.5	13.77	6.4912
	*p* value*, vs. baseline					0.3017			0.835			0.375			0.1209			0.2724		
	SB		3.0891	2.091	0.3745	3.2111	1.784	0.3492	3.2	1.987	0.3611	3.2916	1.742	0.3257	3.6833	2.135	0.7466	3.825	1.226	0.5366
	*p* value*, vs. baseline					0.3508			0.4364			0.9938			0.577			0.2459		
	PCSC		2.8594	3.653	0.643	2.3067	1.554	0.2842	2.4293	1.169	0.225	3.069	3.141	0.5859	2.85	1.741	0.5632	2.4938	6.601	0.4074
	*p* value*, vs. baseline					0.4001			0.5005			0.8053			0.9908			0.5989		
	*p* value among 3 groups**					0.0498			0.062			0.605			0.0421			0.0744		
	*p* value by ANOVA*		0.0460																	
			Baseline			6W			12W			6 M			1Y			2Y		
			Mean	SD	SE	Mean	SD	SE	Mean	SD	SE	Mean	SD	SE	Mean	SD	SE	Mean	SD	SE
mUpH																				
	Standard		5.8581	0.545	0.0963	5.9112	0.511	0.09166	6.0503	0.606	0.1089	5.9187	0.551	0.09741	5.4263	0.641	0.2772	5.8133	0.911	0.4295
	*p* value*, vs. baseline					0.4254			0.959			0.0943			0.0171*			0.2856		
	SB		5.9531	0.505	0.0879	6.0044	0.562	0.1062	6.3119	0.552	0.09751	6.321	0.507	0.08959	6.305	0.168	0.1533	6.4275	0.427	0.08384
	*p* value*, vs. baseline					0.4128			0.2774			0.9335			0.9745			0.9375		
	PCSC		5.8825	0.432	0.0753	6.0497	0.513	0.09211	6.0828	0.556	0.1014	6.2355	0.572	0.1045	6.226	0.594	0.1713	6.3367	0.593	0.1865
	*p* value*, vs. baseline					0.094			0.0843			0.0102*			0.0657			0.0385*		
	*p* value among 3 groups**					0.5566			0.1359			0.0078*			0.019			0.3544		
	*p* value by ANOVA*		0.4020																	
			Baseline			6W			12W			6 M								
			Mean	SD	SE	Mean	SD	SE	Mean	SD	SE	Mean	SD	SE						
UAE	mg/L																			
	Standard		103.37	282.95	49.99	106.42	291.49	52.32	128.14	384.83	69.08	147.57	352.24	62.24						
	*p* value*, vs. baseline					0.3636			0.1519			0.0739								
	SB		43.094	52.84	9.194	70.915	119.16	22.5	125.74	270.13	47.73	98.136	210.39	37.17						
	*p* value*, vs. baseline					0.0928			0.0325*			0.0687								
	PCSC		102.93	198.02	34.46	85.953	204.09	36.64	75.11	216.53	38.85	77.272	237.95	43.42						
	*p* value*, vs. baseline					0.3746			0.3524			0.4138								
	*p* value among 3 groups**					0.8029			0.6487			0.651								
	*p* value by ANOVA*		0.4199																	
			Baseline			6W			12W			6 M								
			Mean	SD	SE	Mean	SD	SE	Mean	SD	SE	Mean	SD	SE						
UNAG	U/L																			
	Standard		4.3387	3.108	0.5492	3.97	3.526	0.6331	3.58	2.579	0.4661	3.6871	2.818	0.5007						
	*p* value*, vs. baseline					0.4388			0.0919			0.2129								
	SB		4.375	3.623	0.633	5.5481	4.923	0.9297	5.8935	6.441	1.1391	5.3484	4.778	0.8442						
	*p* value*, vs. baseline					0.4509			0.4682			0.6046								
	PCSC		3.1438	2.442	0.4288	3.47	4.407	0.781	3.7621	3.986	0.7286	3.6103	3.894	0.7128						
	*p* value*, vs. baseline					0.67			0.3911			0.5021								
	*p* value among 3 groups**					0.2142			0.1703			0.2001								
	*p* value by ANOVA*		0.1665																	
			Baseline			6W			12W			6 M								
			Mean	SD	SE	Mean	SD	SE	Mean	SD	SE	Mean	SD	SE						
UNGAL	ng/mL																			
	Standard		9.0258	14.47	2.5567	8.15	11.53	2.0704	6.9733	7.75	1.3911	6.5903	8.73	1.5432						
	*p* value*, vs. baseline					0.4193			0.4006			0.2771								
	SB		16.0906	27.46	4.7782	18.2481	33.69	6.363	17.9	32.71	5.7805	14.4839	27.97	4.9431						
	*p* value*, vs. baseline					0.9549			0.6588			0.4712								
	PCSC		7.6938	10.31	1.7951	10.1567	20.84	3.7421	7.7862	10.23	1.8668	8.5897	13.04	2.3808						
	*p* value*, vs. baseline					0.5348			0.9601			0.695								
	*p* value among 3 groups**					0.3123			0.186			0.2828								
	*p* value by ANOVA*		0.8242																	
			Baseline			6W			12W			6 M								
			Mean	SD	SE	Mean	SD	SE	Mean	SD	SE	Mean	SD	SE						
UKIM-1	pg/mL																			
	Standard		1571.11	1434.81	253.51	1522.95	1523.47	273.47	1269.69	1204.98	216.3	1399.25	1468.53	259.47						
	*p* value*, vs. baseline					0.8929			0.1887			0.4986								
	SB		1385.02	1074.02	186.87	1627.36	1374.92	259.66	1587.27	1492.82	263.76	1519.19	1217.95	215.19						
	*p* value*, vs. baseline					0.2446			0.6775			0.6962								
	PCSC		1404.71	1076.11	187.23	1323.09	804.29	144.37	1465.63	1152.16	210.23	1424.42	1095.55	199.9						
	*p* value*, vs. baseline					0.64			0.7801			0.9166								
	*p* value among 3 groups**					0.5416			0.6282			0.9241								
	*p* value by ANOVA*		0.4075																	
			Baseline			6W			12W			6 M								
			Mean	SD	SE	Mean	SD	SE	Mean	SD	SE	Mean	SD	SE						
ULFABP	ng/mL																			
	Standard		4.4161	8.839	1.5653	3.3323	5.742	1.034	5.2197	15.282	2.7457	5.0984	11.397	2.0162						
	*p* value*, vs. baseline					0.9919			0.3111			0.228								
	SB		2.4772	2.569	0.4504	2.9237	2.62	0.5001	3.74	6.224	1.1022	2.9287	3.775	0.669						
	*p* value*, vs. baseline					0.2739			0.0974			0.2375								
	PCSC		3.0812	7.704	1.3435	1.9817	2.277	0.4155	1.7703	1.488	0.2761	1.7717	1.602	0.2939						
	*p* value*, vs. baseline					0.4233			0.3345			0.3314								
	*p* value among 3 groups**					0.237			0.1093			0.0888								
	*p* value by ANOVA*		0.1894																	
			Baseline			6W			12W			6 M			1Y			2Y		
			Mean	SD	SE	Man	SD	SE	Mean	SD	SE	Mean	SD	SE	Mean	SD	SE	Mean	SD	SE
U8IsoP	ng/mgCr																			
	Standard		5.0071	2.405	0.425	4.595	1.907	0.3424	4.8383	2.036	0.3656	5.0023	2.642	0.4668	3.1625 (4)†	2.260	1.1302	5.8000 (3)†	2.260	1.305
	*p* value*, vs. baseline					0.4018			0.3116			0.6832			0.875			0.3907		
	SB		4.9519	2.545	0.4429	4.6863	1.72	0.3249	4.5987	2.187	0.3864	4.7194	1.755	0.3101	5.1770 (7)†	2.095	0.7917	6.9180 (5)†	2.095	0.9367
	*p* value*, vs. baseline					0.6257			0.1649			0.9833			0.4609			0.8125		
	PCSC		5.3294	2.371	0.4125	5.3193	2.957	0.5309	5.7279	2.657	0.492	5.1066	2.474	0.4514	5.4390 (10)†	4.708	1.4889	6.4756 (9)†	3.887	1.2958
	*p* value*, vs. baseline					0.9779			0.2617			0.5017			0.3223			0.0814		
	*p* value among 3 groups**					0.4992			0.1829			0.7452			0.4012			0.8148		
	*p* value by ANOVA*		0.7697																	
			Baseline			6W			12W			6 M			1Y			2Y		
			Mean	SD	SE	Mean	SD	SE	Mean	SD	SE	Mean	SD	SE	Mean	SD	SE	Mean	SD	SE
U8OHdG	ng/mgCr																			
	Standard		5.9613	2.679	0.4734	5.78	2.726	0.4894	5.9533	2.659	0.4774	5.3484	1.945	0.3438	5.1750 (4)†	2.490	1.2449	5.4333 (3)†	2.490	1.4375
	*p* value*, vs. baseline					0.443			0.2575			0.7699			1			1		
	SB		5.6625	1.832	0.3188	6.1704	2.53	0.4779	6.1065	1.805	0.319	5.6645	2.263	0.3998	4.1857 (7)†	2.093	0.791	4.8200 (5)†	2.093	0.936
	*p* value*, vs. baseline					0.0393*			0.0603			0.1104			0.1797			0.375		
	PCSC		7.3500	3.151	0.5482	6.7867	2.862	0.5138	6.6276	3.609	0.6587	6.569	2.169	0.3958	4.6600 (10)†	1.357	0.429	4.7333 (9)†	1.121	0.3738
	*p* value*, vs. baseline					0.1801			0.2004			0.0481*			0.002*			0.0137*		
	*p* value among 3 groups**					0.3629			0.7009			0.0623			0.8148			0.8943		
	*p* value by ANOVA*		0.2948																	
			Baseline			6W			12W			6 M								
			Mean	SD	SE	Mean	SD	SE	Mean	SD	SE	Mean	SD	SE						
UTGFb	ng/mL																			
	Standard		0.00753	0.0043	0.0046	0.00031	0.0009	0.00021	0.00023	0.0008	0.000222	0.00146	0.0033	0.00113						
	*p* value*, vs. baseline					0.2824			0.5693			0.604								
	SB		0.00082	0.0025	0.0006	0.002	0.0016	0.00113	0.00114	0.0005	0.000588	0.00136	0.0007	0.00073						
	*p* value*, vs. baseline					0.571			0.1453			0.3348								
	PCSC		0.00525	0.0030	0.0031	0.00433	0.0021	0.00216	0.00107	0.0027	0.000558	0.00229	0.0011	0.00118						
	*p* value*, vs. baseline					0.799			0.1591			0.3963								
	*p* value among 3 groups**					0.0678			0.1698			0.7939								
	*p* value by ANOVA*		0.1740																	
			Baseline			6W			12W			6 M								
			Mean	SD	SE	Mean	SD	SE	Mean	SD	SE	Mean	SD	SE						
UET1	pg/mL																			
	Standard		0.1239	0.1960	0.0461	0.1286	0.1631	0.03644	0.1038	0.1821	0.04171	0.07965	0.1054	0.02481						
	*p* value*, vs. baseline					0.6998			0.8892			0.1568								
	SB		0.1582	0.2435	0.0519	0.2435	0.5456	0.1137	0.2336	0.5401	0.115	0.3403	0.5815	0.1368						
	*p* value*, vs. baseline					0.3847			0.3517			0.5435								
	PCSC		0.1177	0.1650	0.0344	0.1032	0.1123	0.0245	0.0904	0.1280	0.02646	0.2059	0.2933	0.0672						
	*p* value*, vs. baseline					0.6843			0.4511			0.2415								
	*p* value among 3 groups**					0.4406			0.4767			0.0469*								
	*p* value by ANOVA*		0.4058																	
			Baseline			6W			12W			6 M								
			Mean	SD	SE	Mean	SD	SE	Mean	SD	SE	Mean	SD	SE						
UANG	ng/mL																			
	Standard		13.654	26.9939	4.8456	15.3507	28.1011	5.1275	13.1971	32.6308	6.0555	18.6723	39.4664	7.0844						
	*p* value*, vs. baseline					0.7924			0.5466			0.1467								
	SB		10.3831	8.0235	1.396	13.7767	15.1546	2.862	19.9255	33.5371	5.9255	18.0129	24.3673	4.3053						
	*p* value*, vs. baseline					0.5701			0.0631			0.0623								
	PCSC		16.3872	30.1309	5.2426	16.7503	35.6251	6.3949	12.9528	25.0701	4.5052	13.1597	27.6246	5.0405						
	*p* value*, vs. baseline					0.9353			0.4306			0.4692								
	*p* value among 3 groups**					0.898			0.6116			0.7206								
	*p* value by ANOVA*		0.2707																	
			Baseline			6W			12W			6 M								
			Mean	SD	SE	Mean	SD	SE	Mean	SD	SE	Mean	SD	SE						
UMCP-1	pg/mL																			
	Standard		241.71	554.003	98.478	165.78	200.627	36.8316	153.73	116.651	22.3639	151.47	127.306	23.8708						
	*p* value*, vs. baseline					0.1602			0.2644			0.2972								
	SB		179.12	130.299	23.205	193.84	159.055	29.0447	182.61	141.001	25.4446	207.32	165.532	29.6038						
	*p* value*, vs. baseline					0.7242			0.826			0.5719								
	PCSC		154.43	111.531	20.761	185.27	254.635	46.349	166.13	150.869	28.631	159.98	106.646	20.5352						
	*p* value*, vs. baseline					0.3991			0.6935			0.8266								
	*p* value among 3 groups**					0.8356			0.6956			0.3038								
	*p* value by ANOVA*		0.8575																	
			Baseline			6W			12W			6 M								
			Mean	SD	SE	Mean	SD	SE	Mean	SD	SE	Mean	SD	SE						
UIL-6	pg/mL																			
	Standard		2.4541	4.0816	0.7212	2.6655	6.2475	1.1215	1.9381	2.6443	0.4747	2.8118	6.3669	1.1249						
	*p* value*, vs. baseline					0.6533			0.9328			0.589								
	SB		2.9593	5.7737	1.0046	3.2879	4.9873	0.9419	3.2309	4.7339	0.8364	3.2695	5.0758	0.8968						
	*p* value*, vs. baseline					0.5841			0.3386			0.5349								
	PCSC		2.2252	4.7583	0.8279	2.0133	2.9622	0.5317	1.6373	1.7503	0.3143	1.8974	2.3775	0.4338						
	*p* value*, vs. baseline					0.7823			0.4397			0.7197								
	*p* value among 3 groups**					0.4807			0.2024			0.3346								
	*p* value by ANOVA*		0.8900																	
			Baseline			6W			12W			6 M								
			Mean	SD	SE	Mean	SD	SE	Mean	SD	SE	Mean	SD	SE						
UAldo	ng/mL																			
	Standard		4.4516	3.5464	0.6481	4.8933	5.2179	0.9465	3.2667	2.2001	0.4125	3.9903	3.9184	0.7021						
	*p* value*, vs. baseline					0.3998			0.0878			0.3357								
	SB		3.3312	3.5349	0.6316	3.6889	3.3887	0.659	3.5452	4.5962	0.8322	3.1935	2.4645	0.453						
	*p* value*, vs. baseline					0.4723			0.7989			0.4922								
	PCSC		4.3	4.3888	0.7796	3.9133	2.8422	0.5274	4.7828	5.0790	0.9386	4.969	5.7842	1.0698						
	*p* value*, vs. baseline					0.5986			0.5514			0.4844								
	*p* value among 3 groups					0.5661			0.3364			0.2545								
	*p* value by ANOVA		0.3044																	
			Baseline			6W			12W			6 M								
			Mean	SD	SE	Mean	SD	SE	Mean	SD	SE	Mean	SD	SE						
ULac	nmol/μL																			
	Standard		0.123	0.0968	0.0171	0.1188	0.0777	0.01395	0.01184	0.0790	0.01419	0.1107	0.0854	0.0151						
	*p* value*, vs. baseline					0.3374			0.2061			0.0221*								
	SB		0.1469	0.1143	0.0199	0.1935	0.2445	0.04618	0.1365	0.0777	0.01373	0.1681	0.1665	0.02942						
	*p* value*, vs. baseline					0.5318			0.1603			0.3511								
	PCSC		0.1027	0.0611	0.0106	0.1194	0.0931	0.01672	0.1352	0.1289	0.02319	0.1566	0.1146	0.02092						
	*p* value*, vs. baseline					0.1367			0.124			0.0016*								
	*p* value among 3 groups					0.2932			0.6265			0.0898								
	*p* value by ANOVA*		0.1744																	

Values were described mean, standard deviation (SD) and standard error (SE). The significance of comparison vs. baseline was *p < 0.05, and the significance of comparison among the three groups and ANOVA were **p < 0.0167

*SB* sodium bicarbonate; *PCSC* potassium citrate/sodium citrate; *sCr* serum creatinine; *eGFR-Cr* estimated creatinine glomerular filtration rate; *UP* proteinuria; *UaMG* urinary excretion of alfa1-microglobulin; *U4Col* urinary excretion of type-IV collagen; *mUpH* early morning urinary pH; *UAE* urinary excretion of albumin; *UNAG* urinary excretion of N-acetyl-beta-d-glucosaminidase; *UNGAL* urinary excretion of neutrophil gelatinase-associated lipocalin; *UKIM-1* urinary excretion of kidney injury molecule-1; *ULFABP* urinary excretion of L-type fatty acid binding protein; *U8IsoP* urinary excretion of 8-isoprostane; *U8OHdG* urinary excretion of 8-hydroxy-2′-deoxyguanosine; *UTGFb* urinary excretion of transforming growth factor-beta; *UET-1* urinary excretion of endotherin-1; *UANG* urinary excretion of angiotensinogen; *UMCP-1* urinary excretion of monocyte chemotactic protein-1; *UIL-6* urinary excretion of interleukin-6; *UAldo* urinary excretion of aldosterone; *ULac* urinary excretion of lactate

(*N*)†; Number of patients at 1Y and 2Y for the long-term study

### Secondary endpoints

**Fig. 1:**
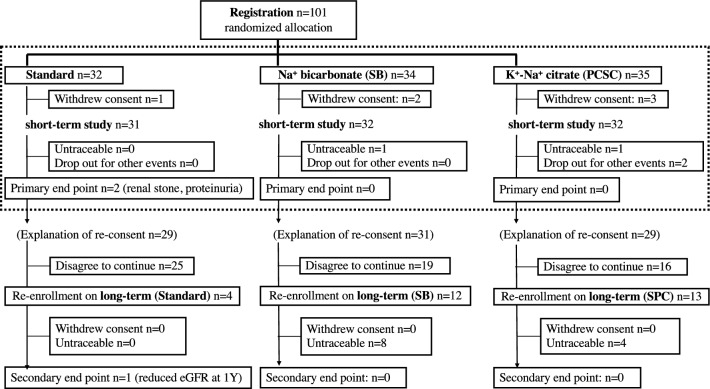
Trial profile of registered patients of the short-term study (dashed-line box) and the long-term study. Patients who finished the short-term study were individually reconsented to continue the long-term study at 6 months

Only 29 patients were included in the long-term study: Standard (*n* = 4), SB (*n* = 12), and PCSC (*n* = 13). Twelve patients in the SB and PCSC groups dropped out because they moved to nearby medical clinics (Fig. 1).

The renal function and urinary surrogate marker results are shown in Table 4. Longitudinal comparisons of sCr and eGFR-Cr showed significant differences among the three groups (*p* = 0.0166 for sCr and *p* = 0.042 for eGFR-Cr by ANOVA). UP significantly increased at 6 M compared with baseline in SB (*p* = 0.0139), but significantly decreased at 1Y and 2Y in PCSC (*p* = 0.0061 and *p* = 0.0186, respectively). The U4Col levels were significantly different among the three groups in the longitudinal comparison (*p* = 0.046). A significant increase in the mUpH was observed at 6 M and 2Y compared with baseline in PCSC (*p* = 0.0102 and *p* = 0.0385, respectively), and there were significant differences at 6 M among the three groups (*p* = 0.0078). Both alkalinizing supplements increased the mUpH. UAE significantly increased at 12W compared to baseline in SB (*p* = 0.0325). 8OHdG significantly increased at 6W in the SB group (*p* = 0.0393) compared to baseline and deceased at 6 M in PCSC (*p* = 0.0481). UET1 at 6 M was significantly different among the three groups (*p* = 0.00469), and both alkalinizing supplements increased the UET1. ULac at 6 M significantly decreased compared to baseline in standard (*p* = 0.0221) and increased in PCSC (*p* = 0.0016). Other urinary surrogate markers were not significantly different among the three groups, and longitudinal comparisons were performed within each group. The SF-8 values after the alkalization of the supplements were not significantly different from those in the standard group (Table 3).

The number of reconsulted patients after the short-term study was small; however, PSCS might suppress urinary excretion of 8OHdG at 1Y and 2Y compared with baseline (*p* = 0.0020 and *p* = 0.0137; Table 4). The most common reason was that taking PCSC made one feel better; however, there were no differences in performance status (Table 3).

## Discussion

We investigated the chronic effects of preserving renal function in mild-stage CKD by neutralizing metabolic acidosis and aciduria (UMIN-CTR 000010059, jRCTs 021180043). We found that a citrate compound of PCSC suppressed the intrarenal ROS. It is also unique that the doses of the alkali loads were adjusted by monitoring the mUpH.

In this study, the progression of renal stones and overt proteinuria occurred in the standard group, but not in the alkalinizing group. PCSC had some reno-protective effects on eGFR-Cr and UP at the secondary endpoints; however, unexpectedly, SB was rather negative. We previously demonstrated that orally administered SB is quickly excreted in the urine and prevents renal injury by suppressing the intrarenal ROS stimulated by both albuminuria and aciduria in vivo [16]. Notably, SB was not effective on the surrogate biomarker for ROS (U8IsoP, 8OHdG, U4Col, UTGFb, and UANG); however, PCSC had protective effects on 8OHdG and U4Col. The relevant suppression of renal ROS by PCSC could be considered as the mechanism of the reno-protective effect. Coincidentally, the results of 8OHdG in the PCSC group tended to be higher than those in the standard and SB groups. The matching factors at registration were age, sex, eGFR, and diabetes status. The urinary biomarkers at baseline could not be matched, and could not be analyzed among the groups. Nevertheless, the reno-protective effects of SB are still controversial. The oral administration of SB or the base produced from FV increased the serum bicarbonate levels and slowed renal dysfunction in patients with CKD with definite metabolic acidosis [14]. Systemic reviews report that SB slowed the decline rate of eGFR [17, 18]. In a series of RCT studies resulting in SB-protected renal functions, the recruited patients had severe stages of CKD with metabolic acidosis [3–5]. In these studies, the inclusion criteria were sufficiently low bicarbonate concentrations (< 21–22 mEq/L), and the participants achieved a target serum bicarbonate concentration of up to 24–28 mmol/L after SB administration. Furthermore, SB improved chronic heart failure and mortality [3] cardiovascular risk [17], and preserved muscle mass [5]. In our study, 8OHdG was significantly increased at 6W and 12W but returned to the basal level at 6 M in the SB group. The intratubular load of high sodium increased the renal oxidative stress from tubular cells by increasing the intracellular transportation of sodium [19, 20] and neutralizing acid conditions of tubules suppressed renal oxidative stress [16]. The effects of SB might be biphasic, sodium loading effects were acutely seen at 6W and 12W, and acid-neutralizing effects by bicarbonate appeared chronically. Thus, SB may have partial reno-protective effects.

We found that the new phenomena that PCSC did not alter 8IsoP, despite a reduction in 8OHdG. A previous study reported that chronic oxidative stress caused by radiation nephropathy increases the urinary excretion of 8-OHdG, but not 8-isoprostane, because they are produced through different pathways, namely DNA oxidation and lipid peroxidation [21]. Another study showed that some citrate-rich fruit extracts stimulated the secretion of prostaglandin E2 in vitro[22]. It is possible that PCSC can suppress renal and/or systemic oxidative stress; however, it simultaneously increases the arachidonic acid levels by citrate. Further studies are needed to test this hypothesis.

To evaluate the effect of proteinuria in patients with CKD associated with urinary excretion of sodium and potassium and the ratio of urinary sodium and potassium excretion (Supplemental Table 1), proteinuria was significantly positively related to sodium and potassium loading only in the SB group, and the effects of urinary sodium or potassium against proteinuria were analyzed (Supplemental Table 2). The reason for this was considered to be that the loading of sodium in the tubules stimulates the production of superoxide anions through the activation of Na/K-ATPase [19]. However, in the PSCS group, the loading of both sodium and potassium was not related to proteinuria. Citrate and/or potassium could attenuate renal injuries caused by sodium but by unknown mechanisms. In future clinical studies, we plan to evaluate the candidate surrogate biomarkers identified in this study precisely.

Patients with severe chronic CKD were excluded. We utilized aciduria (pH < 6.5), including an incision criterion, and the alkali loads were adjusted according to the mUpH levels, but not the serum bicarbonate concentrations. We considered urinary pH as a better index to understand metabolic acidosis because excessive amounts of alkali loads are excreted in the urine [14, 15]. Notably, PCSC significantly increased the mUpH levels but not BS because it was considered that BS was excreted more immediately than PCSC and could not be reflected in the urine the following morning. Additionally, the baseline bicarbonate concentrations in this study were relatively higher (25.7 ± 2.75 mEq/L). Some RCT studies of SB had negative results because patients with low-grade metabolic acidosis (baseline SB of approximately 24 mEq/L) were enrolled [9–11]. This was thought to be the reason why alkali loads are easily buffered and excreted in mild-stage CKD. Nevertheless, PCSC prevented renal function deterioration. In another study, SC preserved the eGFR of cystatin C (CysC) more than eGFR-Cr [12]. A meta-analysis demonstrated that the serum Cr/CysC ratio was positively correlated with muscle mass and strength [23]. The intake of FV-containing citrate was observed to preserve renal function [6, 7, 14]. A notable crossover double-blind study on SC supplementation reported improved tennis performance and reduced fatigue [24]. A different citrate compound, potassium citrate, has been reported to increase serum bicarbonate levels and bone mineral density [25]. Citrate is a major substrate of the tricarboxylic acid cycle in mitochondria, and citrate compounds can metabolically exert organ-protective effects.

## Limitations

This study has several limitations. This was a single-center study that only included Japanese patients, and the enrolled participants were limited to those with mild-CKD stages, including patients with pyuria. After identifying the available surrogate biomarker candidates, they must be precisely addressed and evaluated in future clinical studies. To reveal the reno-protective effects of alkalinizing supplements, we need a larger number of participants for further studies because alkali loads are easily influenced by daily foods and beverages.

## Conclusion

This study is the first to report that the alkalinizing supplementation of PCSC-reduced intrarenal reactive oxygen species in patients with mild-stage CKD. To demonstrate that citrate supplementation prevents the progression of renal dysfunction in patients with CKD, we conducted a cohort study that additionally matched the renal oxidative stress before random stratification.

## Supplementary Information

Below is the link to the electronic supplementary material.

Supplementary file1 (PDF 45 KB)

Supplementary file2 (PDF 30 KB)
