# Correction: Oral alkalinizing supplementation suppressed intrarenal reactive oxidative stress in mild-stage chronic kidney disease: a randomized cohort study

**DOI:** 10.1007/s10157-025-02733-5

**Published:** 2025-07-24

**Authors:** Michiaki Abe, Takuhiro Yamaguchi, Seizo Koshiba, Shin Takayama, Toshiki Nakai, Koichiro Nishioka, Satomi Yamasaki, Kazuhiko Kawaguchi, Masanori Umeyama, Atsuko Masaura, Kota Ishizawa, Ryutaro Arita, Takeshi Kanno, Tetsuya Akaishi, Mariko Miyazaki, Takaaki Abe, Tetsuhiro Tanaka, Tadashi Ishii, CKOALA study group

**Affiliations:** 1 Department of Education and Support for Regional Medicine, Tohoku University Hospital, 1-1 Seiryo-Machi, Sendai, Miyagi 9808574 Japan; 2 Tohoku Medical Megabank Organization, Tohoku University, Sendai, Miyagi Japan; 3 Department of Nephrology, Rheumatology and Endocrinology, Tohoku University Graduate School of Medicine, Sendai, Miyagi Japan; 4 Clinical Research Data Center, Tohoku University Hospital, Sendai, Miyagi Japan; 5 Medical Affairs Department, Nippon Chemiphar Co., Ltd, Chiyoda-Ku, Tokyo, Japan; 6 Development Planning Department, Nippon Chemiphar Co., Ltd, Chiyoda-Ku, Tokyo, Japan; 7 Department of Clinical Biology and Hormonal Regulation, Tohoku University Graduate School of Medicine, Sendai, Miyagi Japan

**Correction: Clinical and Experimental Nephrology (2024) 28:1134–1154** 10.1007/s10157-024-02517-3

In Table 2, the unit for “UAE” was incorrectly stated as “g/gCr.” The correct unit should be “mg/gCr”, and the median value for “UAE” in SB was misreported as “17.9” but should actually be “24.0”.

Additionally, in Table 4, the unit for “UAE” was incorrectly listed as “mg/gCr.” While the numerical values of the UAE level were reported accurately, the correct unit is “mg/L.” The original article has now been corrected.

Here is the revised versions of Table 2 and Table 4:

**Table 2:** Basal characteristics of physiological findings and blood and urine examination on renal function

0w	ALL			Standard			SB			SPC			*p* value*
	*n*	Median	IQ range (Q1, Q3)	*n*	Median	IQ range (Q1, Q3)	*n*	Median	IQ range (Q1, Q3)	*n*	Median	IQ range (Q1, Q3)	
Number	95			31			32			32			
Sex (female)	33			10			11			11			0.9944
Age, years old	95	65	55–70	31	65.0	58, 68	32	65.0	52.3, 70	32	64.0	56.5, 70.8	0.9947
≥ 65	51	70	67, 73	18	68	66, 74.3	17	70	67, 72	16	71	67, 74	0.3817
Physical findings													
Body Height, cm	95	163.5	158.5, 169	31	163.3	155.1, 168.2	32	163.7	159.2, 169.4	31	163.2	158.9, 169.4	0.8287
Body Weight, kg	95	71.5	59.8, 81.5	31	72.9	55, 79.6	32	71.4	58.6, 83.0	32	69.0	60.2, 82.5	0.8842
BMI	95	26.3	22.8, 29.5	31	26.2	23.7, 29.1	32	26.9	23.7, 30.2	32	26.0	22.2, 30.0	0.7373
SBP, mmHg	95	128	119, 139	31	128.0	117, 142	31	132.0	123, 143	32	126.0	115, 134.5	0.1698
DBP, mmHg	95	80	70, 87	31	82.0	78, 86	31	77.0	69, 88	32	74.0	68.3, 85.3	0.2371
Pulse, bpm	95	50	66, 80	31	70.0	64, 79	31	72.0	66, 84	32	76.0	68, 79	0.5458
Blood examinations													
WBC, × 10^3^/uL	95	5.9	4.8, 7	31	5.7	4.8, 6.5	32	6.2	5.1, 7	32	5.8	4.6, 7.4	0.715
RBC, × 10^6^/uL	95	4.62	4.3, 5	31	4.6	4.4, 5.2	32	4.6	4.3, 4.9	32	4.7	4.1, 5.2	0.5986
Hb, g.dL	95	14.2	13.4, 15.5	31	14.2	13.7, 15.4	32	14.2	13.4, 15.1	32	14.4	12.6, 16.4	0.9823
Ht, %	95	42.5	39.9, 46.4	31	42.8	40.6, 46.6	32	41.6	39.7, 45.3	32	43.6	37.4, 47.3	0.7084
Plt, 10^3^/uL	95	212	189, 262	31	229.0	191, 268	32	220.5	191, 269.5	32	200.0	174.5, 240.5	0.0968
Reticulocyte, ‰	91	1.4	1.2, 1.7	29	1.4	1.2, 1.4	30	1.5	1.3, 2	32	1.4	1.2, 1.6	0.9648
BUN, mg/dL	95	17	15, 22	31	19.0	15, 23	32	16.0	14.3, 20	32	16.5	14.3, 22	0.3432
Cr, mg/dL	95	1.01	0.84, 1.20	31	1.01	0.81, 1.43	32	0.99	0.8, 1.13	32	1.03	0.91, 1.22	0.5798
UA, mg/dL	95	5.9	5, 6.8	31	6.2	5, 6.9	32	5.4	5, 6.65	32	6.0	5.15, 6.8	0.4244
TP, g/dL	94	7.2	6.9, 7.5	31	7.2	7.0, 7.5	32	7.2	7.0, 7.4	32	7.1	6.9, 7.7	0.7828
Alb, g/dL	94	4.2	4.1, 4.4	31	4.2	4.0, 4.4	31	4.2	4.1, 4.4	32	4.1	4.1, 4.4	0.5699
Na, mEq/L	95	140	139, 141	31	139.0	138, 141	32	140.5	139.3, 141	32	140.0	139.3, 142	0.1297
K, mEq/L	95	4.3	4.1, 4.6	31	4.4	4.2, 4.6	32	4.3	4, 4.4	32	4.3	4.1, 4.6	0.1103
Cl, mEq/L	95	104	103, 106	31	104	102, 107	32	104	103, 105	32	105	104, 106.8	0.0237
Ca, mg/dL	95	9.3	9.1, 9.6	31	9.2	9.0, 9.6	32	9.4	9.1, 9.6	32	9.3	9.0, 9.6	0.912
IP, mg/dL	95	3.2	2.8, 3.6	31	3.2	2.7, 3.6	32	3.2	2.8, 3.6	32	3.4	3.0, 3.7	0.4157
Glucose	95	106	96, 118	31	104.0	92, 121	32	107.5	96.3, 122	32	106.5	97, 115	0.7006
HbA1cNGS	94	5.9	5.7, 6.2	31	6.0	5.7, 6.2	31	5.9	5.6, 6.2	32	5.9	5.6, 6.2	0.8734
HCO3-, mEq/L	94	24.85	23.5, 26.5	31	25.4	24.2, 26.9	31	25.4	23.5, 26.8	32	24.2	23.2, 26.2	0.1864
aBE, mEq/L	89	0.1	-1, 1.15	30	0.3	-0.75, 1.25	30	0.4	-0.63, 1.53	29	-0.3	-1.7, 0.73	0.0616
eGFR-Cr, mL/min/1.73m^2^	95	55.22	43.27–63.4	31	54.8	38.0, 64.7	32	55.8	46.9, 64.8	32	56.1	43.3, 61.5	0.6587
Urine examinations													
UpH	93	5.83	5.48–6.19	31	5.7	5.4, 6.2	32	5.9	5.5, 6.3	32	5.9	5.5, 6.2	0.715
UP, g/gCr	91	0.1	0.03, 0.23	31	0.1	0.05, 0.18	31	0.1	0.05, 0.25	31	0.1	0.05, 0.32	0.9318
UAE, mg/gCr	91	24.4	7.1, 159.7	31	18.7	8, 111.8	29	24.0	5.3, 175.9	31	42.9	7.1, 301.4	0.7814
Health QOL													
SF8	94	15.5	13–19	31	16.0	12, 19	32	14.5	11.5, 19	31	16.0	14, 20	0.8687

^*^p < 0.0167 for the significance

*IQ* Internal quartile; *Q1* first quartile; *Q3* third quartile; *BMI* body mass index; *SBP* systolic blood pressure; *DBP* diastolic blood pressure; *WBC* white blood cell; *RBC* red blood cell; *Hb* hemoglobin; *Ht* hematocrit; *Plt* platelet; *BUN* blood urea nitrogen; *Cr* creatinine; *UA* uric acid; *TP* total protein; *Alb* albumin; *aBE* actual base excess; *eGFR-Cr* estimated creatinine glomerular filtration rate of creatinine; *UpH* urinary pH; *UP* proteinuria; *UAE* urinary excretion of albumin

**Table 4:** Outcomes of secondary endpoints of urinary surrogate markers of each visit

			Baseline			6W			12W			6 M			1Y			2Y		
			Mean	SD	SE	Mean	SD	SE	Mean	SD	SE	Mean	SD	SE	Mean	SD	SE	Mean	SD	SE
sCr	g/gL																			
	Standard		1.089	0.34	0.06	1.096	0.316	0.056	1.087	0.334	0.059	1.13	0.351	0.062	1.232	0.194	0.097	1.132	0.130	0.075
	*p* value*, vs. baseline					0.269			0.6411			0.5418			0.056			0.7452		
	SB		0.994	0.239	0.042	1.001	0.219	0.036	0.992	0.235	0.041	0.981	0.237	0.041	0.987	0.127	0.048	0.969	0.098	0.044
	*p* value*, vs. baseline					0.1675			0.5473			0.1938			0.7437			0.3394		
	PCSC		1.054	0.246	0.043	1.098	0.265	0.048	1.067	0.239	0.046	1.075	0.251	0.048	1.061	0.171	0.054	1.079	0.174	0.058
	*p* value*, vs. baseline					0.0612			0.5128			0.3844			0.7971			0.557		
	*p* value among 3 groups**					0.1817			0.3121			0.0989			0.0772			0.1176		
	*p* value by ANOVA*		0.0166*																	
			baseline			6W			12W			6 M			1Y			2Y		
			mean	SD	SE	mean	SD	SE	mean	SD	SE	mean	SD	SE	mean	SD	SE	mean	SD	SE
eGFR-Cr	mL/min/1.73 m^2^																			
	Standard		53.94	15.06	2.66	52.87	13.84	2.42	54.25	16.45	2.91	52.01	16.05	2.84	47.92	9.000	4.5	51.81	5.127	2.96
	*p* value*, vs. baseline					0.7781			0.7627			0.4122			0.0402*			0.9234		
	SB		57.53	13.65	2.38	56.27	12.64	2.05	58.05	15.75	2.72	58.12	13.64	2.35	56.63	6.667	2.52	56.8	4.696	2.1
	*p* value*, vs. baseline					0.8818			0.6186			0.3274			0.2691			0.4118		
	PCSC		53.41	10.78	1.88	51.95	12.85	2.3	53.24	12.11	2.23	52.75	11.51	2.1	55.22	8.601	2.72	51.07	7.080	2.36
	*p* value*, vs. baseline					0.1502			0.8717			0.539			0.2487			0.142		
	*p* value among 3 groups**					0.3309			0.3817			0.1533			0.2406			0.1551		
	*p* value by ANOVA*		0.0420																	
			Baseline			6W			12W			6 M			1Y			2Y		
			Mean	SD	SE	Mean	SD	SE	Mean	SD	SE	Mean	SD	SE	Mean	SD	SE	Mean	SD	SE
UP	g/gCr																			
	Standard		0.271	0.577	0.1	0.331	0.8	0.14	0.33	0.729	0.13	0.367	0.731	0.13	0.192	0.074	0.07	0.241	0.066	0.09
	*p* value*, vs. baseline					0.131			0.2538			0.0594			0.6369			0.3713		
	SB		0.252	0.418	0.07	0.31	0.545	0.09	0.359	0.605	0.11	0.421	0.697	0.12	0.162	0.087	0.05	0.256	4.001	0.06
	*p* value*, vs. baseline					0.0883			0.1232			0.0139*			0.7251			0.0555		
	PCSC		0.306	0.509	0.09	0.272	0.514	0.09	0.28	0.527	0.09	0.287	0.486	0.09	0.198	0.004	0.05	0.241	0.068	0.07
	*p* value*, vs. baseline					0.4307			0.6465			0.678			0.0061*			0.0186*		
	*p* value among 3 groups**					0.9234			0.8463			0.6463			0.8743			0.9841		
	*p* value by ANOVA*		0.2443																	
			Baseline			6W			12W			6 M			1Y			2Y		
			Mean	SD	SE	Mean	SD	SE	Mean	SD	SE	Mean	SD	SE	Mean	SD	SE	Mean	SD	SE
UaMG	mg/L																			
	Standard		4.0365	5.731	1.0126	3.8167	5.704	1.0239	4.7	10.787	1.9364	4.35	7.932	1.4415	5.9333	5.556	2.6206	3.9667	2.845	1.3411
	*p* value*, vs. baseline					0.4671			0.3336			0.4306			0.9192			0.6227		
	SB		3.1625	2.808	0.4886	3.6037	3.685	0.696	3.671	3.668	0.6482	3.8968	3.409	0.6024	1.4333	0.252	0.1186	2.4667	2.021	0.9526
	*p* value*, vs. baseline					0.1884			0.2411			0.1826			0.0989			0.8221		
	PCSC		3.3094	3.870	0.6734	2.5233	2.509	0.4505	2.7207	2.901	0.5294	2.8586	2.829	0.5162	5.58	5.887	2.3548	2.32	1.264	0.5055
	*p* value*, vs. baseline					0.2352			0.3884			0.5058			0.3367			0.243		
	*p* value among 3 groups**					0.2889			0.38			0.333			0.0509			0.5169		
	*p* value by ANOVA*		0.1067																	
			Baseline			6W			12W			6 M			1Y			2Y		
			Mean	SD	SE	Mean	SD	SE	Mean	SD	SE	Mean	SD	SE	Mean	SD	SE	Mean	SD	SE
U4Col	μg/gCr																			
	Standard		3.6935	4.661	0.8294	4.5083	7.019	1.2683	3.4933	2.882	0.5216	2.8532	1.567	0.2916	1.825	0.772	0.3342	10.5	13.77	6.4912
	*p* value*, vs. baseline					0.3017			0.835			0.375			0.1209			0.2724		
	SB		3.0891	2.091	0.3745	3.2111	1.784	0.3492	3.2	1.987	0.3611	3.2916	1.742	0.3257	3.6833	2.135	0.7466	3.825	1.226	0.5366
	*p* value*, vs. baseline					0.3508			0.4364			0.9938			0.577			0.2459		
	PCSC		2.8594	3.653	0.643	2.3067	1.554	0.2842	2.4293	1.169	0.225	3.069	3.141	0.5859	2.85	1.741	0.5632	2.4938	6.601	0.4074
	*p* value*, vs. baseline					0.4001			0.5005			0.8053			0.9908			0.5989		
	*p* value among 3 groups**					0.0498			0.062			0.605			0.0421			0.0744		
	*p* value by ANOVA*		0.0460																	
			Baseline			6W			12W			6 M			1Y			2Y		
			Mean	SD	SE	Mean	SD	SE	Mean	SD	SE	Mean	SD	SE	Mean	SD	SE	Mean	SD	SE
mUpH																				
	Standard		5.8581	0.545	0.0963	5.9112	0.511	0.09166	6.0503	0.606	0.1089	5.9187	0.551	0.09741	5.4263	0.641	0.2772	5.8133	0.911	0.4295
	*p* value*, vs. baseline					0.4254			0.959			0.0943			0.0171*			0.2856		
	SB		5.9531	0.505	0.0879	6.0044	0.562	0.1062	6.3119	0.552	0.09751	6.321	0.507	0.08959	6.305	0.168	0.1533	6.4275	0.427	0.08384
	*p* value*, vs. baseline					0.4128			0.2774			0.9335			0.9745			0.9375		
	PCSC		5.8825	0.432	0.0753	6.0497	0.513	0.09211	6.0828	0.556	0.1014	6.2355	0.572	0.1045	6.226	0.594	0.1713	6.3367	0.593	0.1865
	*p* value*, vs. baseline					0.094			0.0843			0.0102*			0.0657			0.0385*		
	*p* value among 3 groups**					0.5566			0.1359			0.0078*			0.019			0.3544		
	*p* value by ANOVA*		0.4020																	
			Baseline			6W			12W			6 M								
			Mean	SD	SE	Mean	SD	SE	Mean	SD	SE	Mean	SD	SE						
UAE	mg/L																			
	Standard		103.37	282.95	49.99	106.42	291.49	52.32	128.14	384.83	69.08	147.57	352.24	62.24						
	*p* value*, vs. baseline					0.3636			0.1519			0.0739								
	SB		43.094	52.84	9.194	70.915	119.16	22.5	125.74	270.13	47.73	98.136	210.39	37.17						
	*p* value*, vs. baseline					0.0928			0.0325*			0.0687								
	PCSC		102.93	198.02	34.46	85.953	204.09	36.64	75.11	216.53	38.85	77.272	237.95	43.42						
	*p* value*, vs. baseline					0.3746			0.3524			0.4138								
	*p* value among 3 groups**					0.8029			0.6487			0.651								
	*p* value by ANOVA*		0.4199																	
			Baseline			6W			12W			6 M								
			Mean	SD	SE	Mean	SD	SE	Mean	SD	SE	Mean	SD	SE						
UNAG	U/L																			
	Standard		4.3387	3.108	0.5492	3.97	3.526	0.6331	3.58	2.579	0.4661	3.6871	2.818	0.5007						
	*p* value*, vs. baseline					0.4388			0.0919			0.2129								
	SB		4.375	3.623	0.633	5.5481	4.923	0.9297	5.8935	6.441	1.1391	5.3484	4.778	0.8442						
	*p* value*, vs. baseline					0.4509			0.4682			0.6046								
	PCSC		3.1438	2.442	0.4288	3.47	4.407	0.781	3.7621	3.986	0.7286	3.6103	3.894	0.7128						
	*p* value*, vs. baseline					0.67			0.3911			0.5021								
	*p* value among 3 groups**					0.2142			0.1703			0.2001								
	*p* value by ANOVA*		0.1665																	
			Baseline			6W			12W			6 M								
			Mean	SD	SE	Mean	SD	SE	Mean	SD	SE	Mean	SD	SE						
UNGAL	ng/mL																			
	Standard		9.0258	14.47	2.5567	8.15	11.53	2.0704	6.9733	7.75	1.3911	6.5903	8.73	1.5432						
	*p* value*, vs. baseline					0.4193			0.4006			0.2771								
	SB		16.0906	27.46	4.7782	18.2481	33.69	6.363	17.9	32.71	5.7805	14.4839	27.97	4.9431						
	*p* value*, vs. baseline					0.9549			0.6588			0.4712								
	PCSC		7.6938	10.31	1.7951	10.1567	20.84	3.7421	7.7862	10.23	1.8668	8.5897	13.04	2.3808						
	*p* value*, vs. baseline					0.5348			0.9601			0.695								
	*p* value among 3 groups**					0.3123			0.186			0.2828								
	*p* value by ANOVA*		0.8242																	
			Baseline			6W			12W			6 M								
			Mean	SD	SE	Mean	SD	SE	Mean	SD	SE	Mean	SD	SE						
UKIM-1	pg/mL																			
	Standard		1571.11	1434.81	253.51	1522.95	1523.47	273.47	1269.69	1204.98	216.3	1399.25	1468.53	259.47						
	*p* value*, vs. baseline					0.8929			0.1887			0.4986								
	SB		1385.02	1074.02	186.87	1627.36	1374.92	259.66	1587.27	1492.82	263.76	1519.19	1217.95	215.19						
	*p* value*, vs. baseline					0.2446			0.6775			0.6962								
	PCSC		1404.71	1076.11	187.23	1323.09	804.29	144.37	1465.63	1152.16	210.23	1424.42	1095.55	199.9						
	*p* value*, vs. baseline					0.64			0.7801			0.9166								
	*p* value among 3 groups**					0.5416			0.6282			0.9241								
	*p* value by ANOVA*		0.4075																	
			Baseline			6W			12W			6 M								
			Mean	SD	SE	Mean	SD	SE	Mean	SD	SE	Mean	SD	SE						
ULFABP	ng/mL																			
	Standard		4.4161	8.839	1.5653	3.3323	5.742	1.034	5.2197	15.282	2.7457	5.0984	11.397	2.0162						
	*p* value*, vs. baseline					0.9919			0.3111			0.228								
	SB		2.4772	2.569	0.4504	2.9237	2.62	0.5001	3.74	6.224	1.1022	2.9287	3.775	0.669						
	*p* value*, vs. baseline					0.2739			0.0974			0.2375								
	PCSC		3.0812	7.704	1.3435	1.9817	2.277	0.4155	1.7703	1.488	0.2761	1.7717	1.602	0.2939						
	*p* value*, vs. baseline					0.4233			0.3345			0.3314								
	*p* value among 3 groups**					0.237			0.1093			0.0888								
	*p* value by ANOVA*		0.1894																	
			Baseline			6W			12W			6 M			1Y			2Y		
			Mean	SD	SE	Man	SD	SE	Mean	SD	SE	Mean	SD	SE	Mean	SD	SE	Mean	SD	SE
U8IsoP	ng/mgCr																			
	Standard		5.0071	2.405	0.425	4.595	1.907	0.3424	4.8383	2.036	0.3656	5.0023	2.642	0.4668	3.1625 (4)†	2.260	1.1302	5.8000 (3)†	2.260	1.305
	*p* value*, vs. baseline					0.4018			0.3116			0.6832			0.875			0.3907		
	SB		4.9519	2.545	0.4429	4.6863	1.72	0.3249	4.5987	2.187	0.3864	4.7194	1.755	0.3101	5.1770 (7)†	2.095	0.7917	6.9180 (5)†	2.095	0.9367
	*p* value*, vs. baseline					0.6257			0.1649			0.9833			0.4609			0.8125		
	PCSC		5.3294	2.371	0.4125	5.3193	2.957	0.5309	5.7279	2.657	0.492	5.1066	2.474	0.4514	5.4390 (10)†	4.708	1.4889	6.4756 (9)†	3.887	1.2958
	*p* value*, vs. baseline					0.9779			0.2617			0.5017			0.3223			0.0814		
	*p* value among 3 groups**					0.4992			0.1829			0.7452			0.4012			0.8148		
	*p* value by ANOVA*		0.7697																	
			Baseline			6W			12W			6 M			1Y			2Y		
			Mean	SD	SE	Mean	SD	SE	Mean	SD	SE	Mean	SD	SE	Mean	SD	SE	Mean	SD	SE
U8OHdG	ng/mgCr																			
	Standard		5.9613	2.679	0.4734	5.78	2.726	0.4894	5.9533	2.659	0.4774	5.3484	1.945	0.3438	5.1750 (4)†	2.490	1.2449	5.4333 (3)†	2.490	1.4375
	*p* value*, vs. baseline					0.443			0.2575			0.7699			1			1		
	SB		5.6625	1.832	0.3188	6.1704	2.53	0.4779	6.1065	1.805	0.319	5.6645	2.263	0.3998	4.1857 (7)†	2.093	0.791	4.8200 (5)†	2.093	0.936
	*p* value*, vs. baseline					0.0393*			0.0603			0.1104			0.1797			0.375		
	PCSC		7.3500	3.151	0.5482	6.7867	2.862	0.5138	6.6276	3.609	0.6587	6.569	2.169	0.3958	4.6600 (10)†	1.357	0.429	4.7333 (9)†	1.121	0.3738
	*p* value*, vs. baseline					0.1801			0.2004			0.0481*			0.002*			0.0137*		
	*p* value among 3 groups**					0.3629			0.7009			0.0623			0.8148			0.8943		
	*p* value by ANOVA*		0.2948																	
			Baseline			6W			12W			6 M								
			Mean	SD	SE	Mean	SD	SE	Mean	SD	SE	Mean	SD	SE						
UTGFb	ng/mL																			
	Standard		0.00753	0.0043	0.0046	0.00031	0.0009	0.00021	0.00023	0.0008	0.000222	0.00146	0.0033	0.00113						
	*p* value*, vs. baseline					0.2824			0.5693			0.604								
	SB		0.00082	0.0025	0.0006	0.002	0.0016	0.00113	0.00114	0.0005	0.000588	0.00136	0.0007	0.00073						
	*p* value*, vs. baseline					0.571			0.1453			0.3348								
	PCSC		0.00525	0.0030	0.0031	0.00433	0.0021	0.00216	0.00107	0.0027	0.000558	0.00229	0.0011	0.00118						
	*p* value*, vs. baseline					0.799			0.1591			0.3963								
	*p* value among 3 groups**					0.0678			0.1698			0.7939								
	*p* value by ANOVA*		0.1740																	
			Baseline			6W			12W			6 M								
			Mean	SD	SE	Mean	SD	SE	Mean	SD	SE	Mean	SD	SE						
UET1	pg/mL																			
	Standard		0.1239	0.1960	0.0461	0.1286	0.1631	0.03644	0.1038	0.1821	0.04171	0.07965	0.1054	0.02481						
	*p* value*, vs. baseline					0.6998			0.8892			0.1568								
	SB		0.1582	0.2435	0.0519	0.2435	0.5456	0.1137	0.2336	0.5401	0.115	0.3403	0.5815	0.1368						
	*p* value*, vs. baseline					0.3847			0.3517			0.5435								
	PCSC		0.1177	0.1650	0.0344	0.1032	0.1123	0.0245	0.0904	0.1280	0.02646	0.2059	0.2933	0.0672						
	*p* value*, vs. baseline					0.6843			0.4511			0.2415								
	*p* value among 3 groups**					0.4406			0.4767			0.0469*								
	*p* value by ANOVA*		0.4058																	
			Baseline			6W			12W			6 M								
			Mean	SD	SE	Mean	SD	SE	Mean	SD	SE	Mean	SD	SE						
UANG	ng/mL																			
	Standard		13.654	26.9939	4.8456	15.3507	28.1011	5.1275	13.1971	32.6308	6.0555	18.6723	39.4664	7.0844						
	*p* value*, vs. baseline					0.7924			0.5466			0.1467								
	SB		10.3831	8.0235	1.396	13.7767	15.1546	2.862	19.9255	33.5371	5.9255	18.0129	24.3673	4.3053						
	*p* value*, vs. baseline					0.5701			0.0631			0.0623								
	PCSC		16.3872	30.1309	5.2426	16.7503	35.6251	6.3949	12.9528	25.0701	4.5052	13.1597	27.6246	5.0405						
	*p* value*, vs. baseline					0.9353			0.4306			0.4692								
	*p* value among 3 groups**					0.898			0.6116			0.7206								
	*p* value by ANOVA*		0.2707																	
			Baseline			6W			12W			6 M								
			Mean	SD	SE	Mean	SD	SE	Mean	SD	SE	Mean	SD	SE						
UMCP-1	pg/mL																			
	Standard		241.71	554.003	98.478	165.78	200.627	36.8316	153.73	116.651	22.3639	151.47	127.306	23.8708						
	*p* value*, vs. baseline					0.1602			0.2644			0.2972								
	SB		179.12	130.299	23.205	193.84	159.055	29.0447	182.61	141.001	25.4446	207.32	165.532	29.6038						
	*p* value*, vs. baseline					0.7242			0.826			0.5719								
	PCSC		154.43	111.531	20.761	185.27	254.635	46.349	166.13	150.869	28.631	159.98	106.646	20.5352						
	*p* value*, vs. baseline					0.3991			0.6935			0.8266								
	*p* value among 3 groups**					0.8356			0.6956			0.3038								
	*p* value by ANOVA*		0.8575																	
			Baseline			6W			12W			6 M								
			Mean	SD	SE	Mean	SD	SE	Mean	SD	SE	Mean	SD	SE						
UIL-6	pg/mL																			
	Standard		2.4541	4.0816	0.7212	2.6655	6.2475	1.1215	1.9381	2.6443	0.4747	2.8118	6.3669	1.1249						
	*p* value*, vs. baseline					0.6533			0.9328			0.589								
	SB		2.9593	5.7737	1.0046	3.2879	4.9873	0.9419	3.2309	4.7339	0.8364	3.2695	5.0758	0.8968						
	*p* value*, vs. baseline					0.5841			0.3386			0.5349								
	PCSC		2.2252	4.7583	0.8279	2.0133	2.9622	0.5317	1.6373	1.7503	0.3143	1.8974	2.3775	0.4338						
	*p* value*, vs. baseline					0.7823			0.4397			0.7197								
	*p* value among 3 groups**					0.4807			0.2024			0.3346								
	*p* value by ANOVA*		0.8900																	
			Baseline			6W			12W			6 M								
			Mean	SD	SE	Mean	SD	SE	Mean	SD	SE	Mean	SD	SE						
UAldo	ng/mL																			
	Standard		4.4516	3.5464	0.6481	4.8933	5.2179	0.9465	3.2667	2.2001	0.4125	3.9903	3.9184	0.7021						
	*p* value*, vs. baseline					0.3998			0.0878			0.3357								
	SB		3.3312	3.5349	0.6316	3.6889	3.3887	0.659	3.5452	4.5962	0.8322	3.1935	2.4645	0.453						
	*p* value*, vs. baseline					0.4723			0.7989			0.4922								
	PCSC		4.3	4.3888	0.7796	3.9133	2.8422	0.5274	4.7828	5.0790	0.9386	4.969	5.7842	1.0698						
	*p* value*, vs. baseline					0.5986			0.5514			0.4844								
	*p* value among 3 groups					0.5661			0.3364			0.2545								
	*p* value by ANOVA		0.3044																	
			Baseline			6W			12W			6 M								
			Mean	SD	SE	Mean	SD	SE	Mean	SD	SE	Mean	SD	SE						
ULac	nmol/μL																			
	Standard		0.123	0.0968	0.0171	0.1188	0.0777	0.01395	0.01184	0.0790	0.01419	0.1107	0.0854	0.0151						
	*p* value*, vs. baseline					0.3374			0.2061			0.0221*								
	SB		0.1469	0.1143	0.0199	0.1935	0.2445	0.04618	0.1365	0.0777	0.01373	0.1681	0.1665	0.02942						
	*p* value*, vs. baseline					0.5318			0.1603			0.3511								
	PCSC		0.1027	0.0611	0.0106	0.1194	0.0931	0.01672	0.1352	0.1289	0.02319	0.1566	0.1146	0.02092						
	*p* value*, vs. baseline					0.1367			0.124			0.0016*								
	*p* value among 3 groups					0.2932			0.6265			0.0898								
	*p* value by ANOVA*		0.1744																	

Values were described mean, standard deviation (SD) and standard error (SE). The significance of comparison vs. baseline was *p < 0.05, and the significance of comparison among the three groups and ANOVA were **p < 0.0167

*SB* sodium bicarbonate; *PCSC* potassium citrate/sodium citrate; *sCr* serum creatinine; *eGFR-Cr* estimated creatinine glomerular filtration rate; *UP* proteinuria; *UaMG* urinary excretion of alfa1-microglobulin; *U4Col* urinary excretion of type-IV collagen; *mUpH* early morning urinary pH; *UAE* urinary excretion of albumin; *UNAG* urinary excretion of N-acetyl-beta-d-glucosaminidase; *UNGAL* urinary excretion of neutrophil gelatinase-associated lipocalin; *UKIM-1* urinary excretion of kidney injury molecule-1; *ULFABP* urinary excretion of L-type fatty acid binding protein; *U8IsoP* urinary excretion of 8-isoprostane; *U8OHdG* urinary excretion of 8-hydroxy-2′-deoxyguanosine; *UTGFb* urinary excretion of transforming growth factor-beta; *UET-1* urinary excretion of endotherin-1; *UANG* urinary excretion of angiotensinogen; *UMCP-1* urinary excretion of monocyte chemotactic protein-1; *UIL-6* urinary excretion of interleukin-6; *UAldo* urinary excretion of aldosterone; *ULac* urinary excretion of lactate

(*N*)†; Number of patients at 1Y and 2Y for the long-term study

