# Supplementary material for: Oral alkalinizing supplementation suppressed intrarenal reactive oxidative stress in mild-stage chronic kidney disease: a randomized cohort study
Source: Clin Exp Nephrol. 2024 Jun 13;28(11):1134–54. doi: 10.1007/s10157-024-02517-3 (PMC11568046; doi:10.1007/s10157-024-02517-3)
Supplement: Supplementary file 1 — Supplementary file1 (PDF 45 KB) [file 10157_2024_2517_MOESM1_ESM.pdf]

		baseline			6W			12W			6M			1Y			2Y		
		mean	SD	SE	mean	SD	SE	mean	SD	SE	mean	SD	SE	mean	SD	SE	mean	SD	SE
UNa (mEq/gCr)	Standard (31)	154.0	93.5	16.8	137.4	62.4	11.2	157.0	85.7	15.4	146.1	76.3	13.7	82.3 (4)†	44.2	22.1	207.3 (3)†	69.8	40.3
	p value*, vs. baseline	-			0.448			0.411			0.158			0.0077*			0.922		
	SB (31)	153.6	79.1	14.2	161.7	81.3	14.6	193.5	123.6	22.2	177.7	106.3	19.1	112.4 (7)†	49.7	18.8	132.5 (5)†	943.8	422.1
	p value*, vs. baseline	-			0.808			0.58			0.76			0.042			0.196		
	PCSC (32)	131.7	107.1	17.5	134.4	72.1	13.2	157.4	72.6	13.7	164.5	93.7	17.4	146.1 (10)†	61.3	19.4	177.5 (9)†	117.3	39.1
	p value*, vs. baseline	-			0.881			0.163			0.139			0.506			0.305		
	p value among 3 groups**	-			0.319			0.338			0.382			0.908			0.718		
	p value by ANOVA*	0.0115*																	

		baseline			6W			12W			6M			1Y			2Y		
		mean	SD	SE	mean	SD	SE	mean	SD	SE	mean	SD	SE	mean	SD	SE	mean	SD	SE
UK (mEq/gCr)	Standard (31)	53.7	31.8	5.71	51.0	25.1	4.51	53.1	24,4	4.39	51.1	23.1	4.14	40.6 (4)†	11.6	5.79	49.6 (3)†	17.5	10.1
	p value*, vs. baseline	-			0.202			0.303			0.014			0.531			0.318		
	SB (31)	45.7	18.7	3.36	46.6	21.8	3.92	49.0	26.5	4.76	48.3	29.0	5.20	25.2 (7)†	15.4	5.82	48.0 (5)†	10.1	4.48
	p value*, vs. baseline	-			0.530			0.663			0.170			0.142			0.548		
	PCSC (32)	52.8	28.1	4.97	56.1	26.6	4.71	59.1	38.9	6.87	63.4	41.6	7.35	45.0 (10)†	13.7	4.33	58.4 (9)†	1.94	6.48
	p value*, vs. baseline	-			0.198			0.284			0.170			0.142			0.548		
	p value among 3 groups**	-			0.314			0.372			0.117			0.124			0.06		
	p value by ANOVA*	0.1627																	

		baseline			6W			12W			6M			1Y			2Y		
		mean	SD	SE	mean	SD	SE	mean	SD	SE	mean	SD	SE	mean	SD	SE	mean	SD	SE
UNa/K ratio	Standard (31)	3.17	1.89	0.34	2.90	1.22	0.22	3.16	1.69	0.21	2.95	1.17	0.21	2.05 (4)†	1.10	0.55	2.21 (3)†	0.19	0.11
	p value*, vs. baseline	-			0.740			0.272			0.366			0.066			0.057		
	SB (31)	3.46	1.74	0.313	3.72	1.89	0.34	4.44	2.56	0.46	4.09	2.51	0.45	3.53 (7)†	0.77	0.29	2.86 (5)†	1.32	0.59
	p value*, vs. baseline	-			0.265			0.297			0.374			0.102			0.738		
	PCSC (32)	2.57	1.47	0.26	2.37	0.90	0.16	3.06	1.75	0.31	2.7	1.41	0.25	2.95 (10)†	1.87	0.59	3.10 (9)†	1.80	0.6
	p value*, vs. baseline	-			0.562			0.135			0.531			0.054			0.794		
	p value among 3 groups**	-			0.003**			0.031			0.035			0.630			0.003*		
	p value by ANOVA*	0.0951																	

Supplemental Table 1. Additional data of urinary excretion of Na, and K, and urinary Na/K ratio. Values were described mean, standard deviation (SD) and standard error (SE).
The significance of comparison vs. baseline was defined as *p<0.05, and the significance of comparison among the three groups and ANOVA were **p<0.0167.

(N) †; Number of patients at 1Y or 2Y for the long-term study.
