# Supplementary material for: Oral alkalinizing supplementation suppressed intrarenal reactive oxidative stress in mild-stage chronic kidney disease: a randomized cohort study
Source: Clin Exp Nephrol. 2024 Jun 13;28(11):1134–54. doi: 10.1007/s10157-024-02517-3 (PMC11568046; doi:10.1007/s10157-024-02517-3)
Supplement: Supplementary file 2 — Supplementary file2 (PDF 30 KB) [file 10157_2024_2517_MOESM2_ESM.pdf]

		Standard			SB			PCSC		
		UNa vs. UP	UK vs. UP	UNa/K vs. UP	UNa vs. UP	UK vs. UP	UNa/K vs. UP	UNa vs. UP	UK vs. UP	UNa/K vs. UP
0W										
	Slope	-0.0003	-0.0022	0.0106	0.0013	0.0072	-0.0063	-0.0003	-0.0019	-0.0013
	r2	0.003	0.015	0.001	0.060	0.108	0.001	0.003	0.012	0.000
	p	0.781	0.516	0.853	0.184	0.071	0.883	0.785	0.544	0.984
6W										
	Slope	0.0009	-0.0065	0.2275	0.0021	0.0096	0.0138	-0.0002	-0.0020	0.0599
	r2	0.006	0.034	0.123	0.093	**0.153**	0.002	0.001	0.011	0.011
	p	0.689	0.338	0.062	0.121	**0.044***	0.820	0.892	0.582	0.586
12W										
	Slope	0.0012	-0.0041	0.1622	0.0013	0.0034	0.0332	-0.0003	-0.0019	0.0257
	r2	0.019	0.018	0.108	0.081	0.024	0.020	0.002	0.019	0.006
	p	0.458	0.466	0.071	0.121	0.410	0.450	0.840	0.491	0.698
6M										
	Slope	0.0013	-0.0021	0.1808	0.0029	0.0148	-0.0169	0.00001	-0.0007	0.0447
	r2	0.019	0.005	0.085	**0.211**	**0.397**	0.004	0.000	0.003	0.015
	p	0.464	0.717	0.112	**0.009***	**0.0001***	0.744	0.990	0.767	0.524

Supplemental Table 2. Comparison between proteinuria and urinary excretion of sodium or potasseium, or the ratio of urinary sodium and potassium excretion. The significant values were defined by bold (**p*<0.05).
